# Long-Standing International Cooperation in Parasitology Research: A Summary of 35 Years of Activities in the Bolivian Chaco

**DOI:** 10.3390/tropicalmed7100275

**Published:** 2022-09-29

**Authors:** Simona Gabrielli, Fabio Macchioni, Michele Spinicci, Marianne Strohmeyer, Mimmo Roselli, Alessandra Nicoletti, Calogero Edoardo Cicero, Veronica Poma, David Rojo, Yunni Lara, Elizabeth Blanca Crespo Gómez, Patricia Rojas, Herlan Gamboa, Ana Liz Villagran, Francesco Cosmi, Joaquín Monasterio, Gabriella Cancrini, Alessandro Bartoloni

**Affiliations:** 1 Dipartimento di Sanità Pubblica e Malattie Infettive, Sapienza Università di Roma, 00185 Rome, Italy; 2 Dipartimento di Scienze Veterinarie, Università di Pisa, 56124 Pisa, Italy; 3 Dipartimento Medicina Sperimentale e Clinica, Università degli Studi di Firenze, 50121 Florence, Italy; 4 Dipartimento di Scienze Mediche, Chirurgiche e Tecnologie Avanzate G.F. Ingrassia, Sez. di Neuroscienze, Università di Catania, 95125 Catania, Italy; 5 Escuela de Salud del Chaco Tekove Katu, Gutierrez, Bolivia; 6 Hospital San Antonio de los Sauces, Monteagudo, Bolivia; 7 Hospital Hernández Vera, Santa Cruz, Bolivia; 8 Caja Petrolera Hospital, Camiri, Bolivia; 9 Facultad Integral del Chaco, Universidad Autónoma Gabriel René Moreno, Camiri, Bolivia; 10 Hospital Básico Villa Montes, Villa Montes, Bolivia; 11 Convenio de Salud, Camiri, Bolivia; 12 Servicio Departamental de Salud (SEDES) de Santa Cruz, Santa Cruz, Bolivia

**Keywords:** Bolivia, NTDs, soil-transmitted helminths, parasitology, epidemiology

## Abstract

The Bolivian Chaco is a semiarid region with a low population density, situated in the southeast part of the Plurinational State of Bolivia. Here, despite the improvements of the last 15 years, poverty remains high in rural areas, where social vulnerability is widespread. The Guaraní ethnic group often lives in isolated communities with a low standard of hygiene and sanitation. This epidemiological scenario favors the spread of transmissible diseases, including several parasitic infections belonging to the neglected tropical diseases (NTDs) group. In this area, a long-standing research activity, built upon the synergism between local and foreign institutions, has been established since the late 1980s and helps to fill in the knowledge gap about the epidemiology dynamics of soil-transmitted helminths, vector-borne parasites, and other parasitic diseases. A 35-year history of cooperation programs in parasitology research has contributed to informing local health authorities of the NTD burden in the Bolivian Chaco and, ultimately, supports local healthcare providers in the management of parasitic diseases.

## 1. Introduction

**Figure 1:**
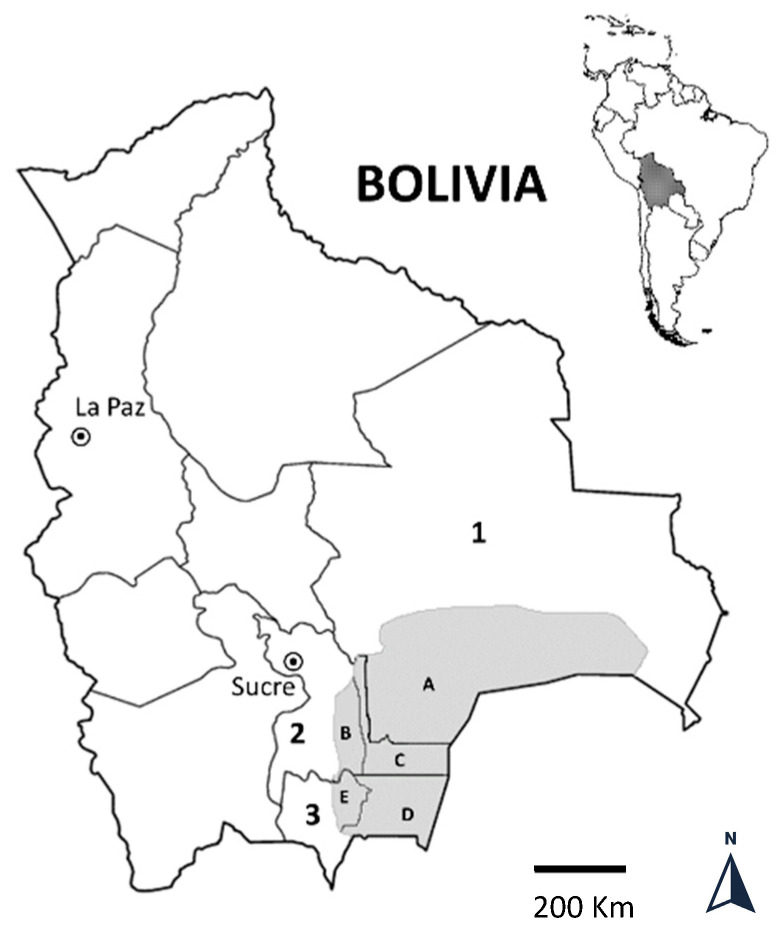
The area of the Bolivian Chaco lies in the southeast of the country and includes 3 departments (numbers on the map: Santa Cruz = 1; Chuquisaca = 2; Tarija = 3) and 5 Provinces (letters on the map: Cordillera = A; Hernando Siles = B; Luis Calvo = C; Gran Chaco = D; O’Connor = E).

The Bolivian Chaco region is a semiarid and homogeneous ecological zone, located between the latitudes 17°59′–22°21′ south and the longitudes 64°31′–58°51′ west. The region, approximately 127,755 km^2^ in size, has few inhabitants and comprises three departments (Chuquisaca, Santa Cruz, and Tarija) and five provinces (Gran Chaco, Luis Calvo, Cordillera, Hernando Siles, and O’Connor) (Figure 1). Over the past 15 years, Bolivia has made significant economic and social advances, with a drastic reduction in extreme poverty from 38.2% in 2005 to 12.9% in 2019, with a setback to 39% in 2020 caused by the COVID-19 pandemic [1]. The majority of the population, mostly of Guarani ethnicity, still live in a few small urban centers, where households live in poor houses, with walls of sticks, straw, and clay, and the roofs are generally made of sheet iron or thatch. People living in isolated rural communities do not achieve an adequate level of hygiene and sanitation. Their principal economy depends on agriculture and livestock production.

This epidemiological setting favors the spread of several infectious diseases affecting the populations of low- and middle-income countries (LMIC). Most of them belong to the so-called “neglected tropical diseases” (NTDs) group, which tend to thrive in developing countries, where the water quality, sanitation, and access to healthcare are below the standards.

Over the last 35 years, long-standing research activity has been conducted in the Bolivian Chaco by local health institutions and international partners in the field of communicable and noncommunicable diseases with an official research and cooperation agreement signed in 1987 between the University of Florence and the Bolivian Ministry of Health (MoH). These projects aimed to support the development of basic health services; promote sanitation; and help the local sanitary personnel in the prevention, control, diagnosis, and treatment of infectious diseases. Throughout these years, the activities have grown continuously, with the progressive inclusion of several Italian, European, and Latin American institutions.

Since the beginning, parasite infections—including soil-transmitted helminths (STHs), vector-borne parasites, and other parasitic diseases—have represented a focal point of the cooperation projects. Here, we report the main findings of the research activities conducted within the cooperation agreement in the last 35 years in the field of parasitic diseases.

## 2. Intestinal Parasitic Infections

Intestinal parasitic infections (IPIs) are amongst the most common parasites affecting the poorest and most deprived communities of tropical LMIC and are included on the World Health Organization (WHO) list of NTDs [2]. STHs such as *Ascaris lumbricoides*, *Trichuris trichiura*, and hookworms are the most widespread intestinal parasites, affecting about 24% of the world’s population, with 135,000 deaths estimated annually [2]. Among protozoa, *Giardia intestinalis* (syn. *G. lamblia*–*G. duodenalis*) is the most common species, with an estimated 1.2–2 million cases per year and infection rates from 0.4 to 7.6% in developed countries and from 0.9 to 40% in LMIC [3]. Although they are rarely the direct cause of death, IPIs can induce malabsorption, diarrhea, blood loss, anemia, reduced work capacity, and reduced growth, thus representing important health and social problems.

In the Bolivian Chaco, studies conducted in rural communities of the Santa Cruz Department in the 1980s and 1990s detected an STH prevalence from 64% to 41% [4,5,6].

**Figure 2:**
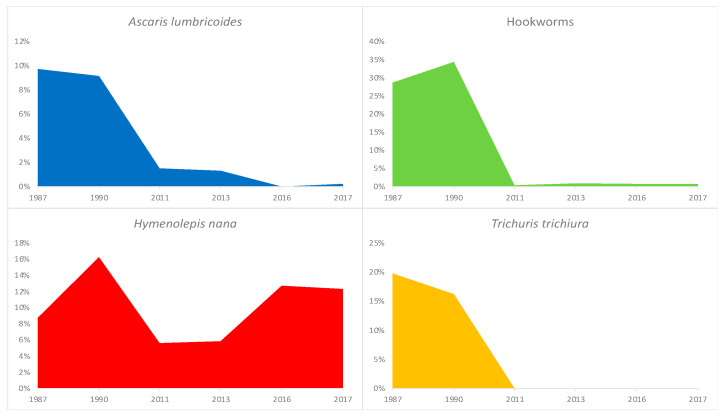
Soil-transmitted helminths trend during 1987–2017 in the Bolivian Chaco. A *Hymenolepis nana* trend was also shown as an example of the feco–oral-transmitted parasite. The study population constituted a general population in 1987 (*n* = 381) [5] and 2013 (*n* = 223) [9], pre-school- and school-aged children (SAC) in 1990 (*n* = 179) [10] and 2011 (*n* = 268) [7], and only SAC in 2016 (*n* = 426) and 2017 (*n* = 520) [10]. In 1987 and 2011, the surveyed areas included both rural and urban areas, while other surveys were limited to rural areas.

Notably, in 1985, the Bolivian MoH launched a mass deworming program using a single-dose administration system of mebendazole for school-aged children (SAC) [7]. Given that the epidemiology of STHs is likely to change after several cycles of drug administration, parasitological evaluations should be repeated periodically in the areas of intervention and the frequency of PC (preventive chemotherapy) administration adjusted accordingly [8]. Further parasitological surveys conducted in rural and urban areas of the Bolivian Chaco two to three decades later (2011 and 2013) showed an impressive reduction in the prevalence of STH infection; compared with results from 1987, the prevalence of hookworms decreased from 50% to 0.4–1.3%; of *A. lumbricoides*, from 19% to 0.9–1.5%; and of *T. trichiura*, from 19% to 0% [5,7,9] (Figure 2). According to these findings documenting a low prevalence of STHs in September 2016, the local Departmental Health Services (SEDES) halted the delivery of PC in the Bolivian Chaco and launched a STH monitoring program through annual cross-sectional parasitological surveys at sentinel sites to monitor the effects of reduced PC on STH transmission [10].

The results from cross-sectional surveys performed in 2016 and 2017 amongst SAC living in such sentinel sites showed an STH prevalence of 0.7% in 2016 and 0.8% in 2017. The same studies showed a high prevalence, comparable to that of the 1980s, of infections with the tapeworm *H. nana* (13% in 2016, 12% in 2017) and intestinal protozoan (81% in 2016, 75% in 2017), suggesting that environmental fecal contamination from inadequate sanitation, poor hygiene, and unsafe drinking water persists in the Bolivian Chaco (Figure 2). Thus, these results support the role of PC in reducing the transmission of STH. Remarkably, this dramatic decline does not seem to have changed the risk of anemia in school-aged children over the last three decades. Children who participated in cross-sectional parasitology surveys were additionally subjected to a single Hb measurement using a portable Hb analysis system: about one in four to one in five children were anemic, half of them with moderate anemia (Hb from 11 to 8 g/dL). The situation has not improved over the past quarter-century. Indeed, a study conducted in 1990 in two rural communities in the same region showed 22% and 16% and a mean Hb of 11.8 and 12.4 g/dL among pre-SAC (2–5 years old) and SAC (6–9 years old), respectively [11]. Interestingly, in the population from the 2016 survey, *H. nana* infection was associated with a three-fold greater risk of anemia [12].

## 3. Malaria

Malaria is endemic in remote parts of the Amazon region of Bolivia, while the areas with the highest altitude are low and mostly unstable. *Plasmodium vivax* represents the species most prevalent in 93% of the reports, while the remaining 7% show *Plasmodium falciparum* [13]. In general, in recent years, the incidence of malaria has fallen (from 30,126 cases in 2000 to 7342 in 2013), and the country has not recorded any deaths from malaria since 2004. The decrease in cases relates to the prevention measures, including vector–control strategies, which have been implemented since 2003 at the national level [14].

Between 1988 and 1989, two cross-sectional surveys were conducted to assess the prevalence of malaria in eight rural villages (Santa Cruz Department). Blood samples were collected from 252 children aged two to nine at the end of the dry season and, in the second investigation, from 346 subjects after the rainy season. The parasite and gametocyte indices increased between surveys from 1·59 to 25·72 and from 0·40 to 1·73, respectively. The prevalence and parasitic load were lower in children aged two to four than in older children and generally highest in rural areas [15].

## 4. Babesia

Taking advantage of studies addressing malaria epidemiology, in 2013, blood samples were collected from 271 healthy people in two rural communities in this region. Surprisingly, *Babesia microti* parasites (US lineage) were identified by microscopy and PCR in 3.3% of enrolled subjects, with a general seroprevalence of 45.7%, highlighting the human exposure to ticks and tick-borne diseases transmitted from wild animals [16]. Of note, *Plasmodium* spp. were not detected in any sample from the same population. Moreover, the results from this report also highlighted the possibility that babesiosis may contribute to anemia in the studied populations.

## 5. Filariasis

The human filarial *Mansonella ozzardi* is widely present in the Neotropical region from Southern Mexico to Northwest Argentina. It causes mansonelloses and is transmitted by black flies of the genus *Simulium* and stinging midges of the family Ceratopogonidae. [17].

In Bolivia, the first symptomatic case of *M. ozzardi* was reported in Santa Cruz [18]. In addition, the parasite was occasionally detected in thick blood smears collected during a routine malaria investigation (Vallejos and Lagrava, unpublished data) and patients who admitted to Camiri District Hospital. [18]. A cross-sectional survey carried out in the Chaco region showed that *M. ozzardi* microfilariae (mf) were present in 26% (77 out of 296) and 0.7% (2 out of 298) of the rural population from Camiri and Villa Montes, respectively. No significant differences were observed between the sexes. No children under the age of 11 months were infected, and the age group with the lowest prevalence (9%) was 0–14 years old. The prevalence increased significantly (to 32%) in the 25–34-year-old age group and kept rising as people aged. The microfilaremia mean (51.1 mf/20 mL), ranging from 1 to 305 mf/20 mL of blood, was lower in children aged 0–14, and it increased with age (100 mf/20 mL in those over 44 years old). An expected increasing sensitivity with the blood volume examined was observed. There was no discernible connection between microfilaremia and any of the clinical symptoms, including fever, rash, pruritus, headache, lymphedema, elephantiasis, and articular pain [19].

As far as zoonotic dirofilariasis is concerned, a study was conducted to evaluate the presence of zoonotic dirofilariasis in the dog population of the Chaco region, where more than 90% of the households owned a domestic dog. In the Americas, the principal agent of canine dirofilariasis is *Dirofilaria immitis*, and in Bolivia, this was detected in dogs and in wild canids on the border of the Noel Kempff Mercado National Park [20]. Our preliminary screening carried out on 100 dogs living in the communities of Bartolo and Ivamirapinta revealed antigens of *D. immitis* in 3% of the dogs, confirming the circulation of *D. immitis* in the canine population in the Chaco region [21]. To our knowledge, no clinical cases of human dirofilariasis have been described in the country to date.

## 6. Chagas Disease

Chagas disease is a serious health problem in several Latin American countries, caused by the parasite *Trypanosoma cruzi*, which is naturally transmitted by triatomine bugs. The disease affects 6 to 7 million individuals worldwide and is endemic in several Latin American countries, where household vector infestation represents the main source of transmission [22,23]. On the other hand, in nonendemic countries such as Europe, infection may occur by blood transfusion or organ transplantation and by transmission from mother to fetus during pregnancy, representing an emerging public health challenge in these areas.

With an estimated 607,186 prevalent infections and an endemic area covering 60% of the nation, Bolivia has the highest incidence rate on the planet (6.1%) [23]. The interruption of vector-borne transmission in several countries of Latin America (Brazil, Chile, Eastern Paraguay, Uruguay, and some provinces of Argentina) was achieved by regional intergovernmental control programs, started in the 1990s, such as the Southern Cone Initiative (1991), the Andean Pact Initiative, and the Central America Initiative (1997) [24]. Such control interventions achieved limited success in the Bolivian Chaco. Indeed, in this area, the risk of transmission remains high, and the home infestation rate is still near 3% in most municipalities of Santa Cruz, Chuquisaca, and Tarija Departments [25]. Indoor spraying campaigns of insecticides were sporadic and/or intermittent in isolated rural communities of the Bolivian Chaco [26], where, in 2000 and 2003, insecticide campaigns were carried through blanket spraying using 20% alpha-cypermethrin, followed by focal spraying on infested houses from 2005 to 2009 and unsystematic spraying from 2009 to 2011 [27]. The low efficacy of IRS (indoor residual spraying) was attributed to the construction of vulnerable houses, suboptimal spraying practices, the short activity period of insecticides, and the reduction in vector sensitivity. [28]. Housing improvements using local materials to seal crevices in the walls and under the roofs, which provide substantial refuges for triatomines, can reduce infestations and increase the IRS effectiveness against *T. infestans* [28]. Community-based participatory approaches have proven to be synergistic tools for the implementation of vector control practices through large-scale housing improvements [26], despite a recent study evidencing the need for the modification of insecticide delivery methods, including the training of IRS teams and community education [28].

From 1987 to 2013, we conducted four seroprevalence studies for *T. cruzi* antibodies throughout the Bolivian Chaco, including different epidemiological settings [29], showing a partial decline of anti-*T. cruzi* seroprevalence over the past 40 years, though it is difficult to conduct a reliable analysis of the epidemiological trend of Chagas disease.

In detail, in 1987, the exposure to *T. cruzi* in a rural setting was widespread from the time of birth but steadily decreased and became age-dependent after that. In 1997, the seroprevalence in some rural communities was around 80%, but in 2006 and 2013, we observed a slight decline in *T. cruzi* seroprevalence in rural communities, ranging from 69.9% to 60.3% [29]. In all the surveyed populations, the seroprevalence among women of reproductive age (15–45 years) in rural areas of all the populations surveyed remained high, ranging from 100% in 1987 to 74–79% in 2013, suggesting a significant risk of Chagas disease transmission by the vertical route (Table 1).

Recently, the results of a longitudinal *T. cruzi* serosurvey among a cohort of 120 SAC living in rural communities at three time points 12 months apart in the period 2017–2019 evidenced a prevalence of 5.8%, 6.6%, and 9.2% in 2017, 2018, and 2019, respectively, and an average incidence of 1.76 per 100 person/years [30] (Table 1). These findings support the persistence of vector-borne *T. cruzi* transmission in the Bolivian Chaco, highlighting the need to strengthen sustained multidisciplinary efforts, including vector control, clinical care, and culturally appropriate community-based approaches.

Moreover, the presence of multiple animal reservoirs represents a further task for disease control and prevention measures in this region. In fact, as a link between sylvatic and domestic *T. cruzi* transmission cycles, the canine population may be crucial to the peridomestic circulation. The role of the dog as a significant domestic reservoir of *T. cruzi* was validated by a cross-sectional investigation of 105 dogs from rural settlements with very high human endemicity (60%), since 22% of the animals tested were positive [31].

## 7. Cysticercosis, Toxocariasis, and Epilepsy

*Taenia solium* is a zoonotic parasite causing taeniasis in humans (the intestinal dwelling of an adult parasite) and cysticercosis in pigs and humans (tissue infection by the metacestode larval stage). Humans can acquire cysticercosis by accidental ingestion of eggs through food or water contaminated by feces from human tapeworm carriers or by autoinfection. *T. solium* is still endemic in several areas of the world, including most of Latin America, sub-Saharan Africa, Southeast Asia, the Indian subcontinent, and parts of China [32]. In these areas, cysticercosis involves the central nervous system (CNS), causing neurocysticercosis (NCC), which is considered the major cause of neurologic diseases. Clinical manifestations of NCC depend on the location of the parasite within the CNS (parenchymal or extra parenchymal NCC), and although NCC has a wide range of clinical manifestations, seizures are the most frequent ones. Indeed, NCC accounts for about one-third of the cases of epilepsy in an endemic region and is considered the main cause of epilepsy in LMIC [32].

With 0.7% of the world’s illness burden is attributed to epilepsy, it is a serious public health concern everywhere. Around 70 million individuals are affected globally, and most of them reside in LMIC [33]. According to an epidemiological study carried out in 1994 in the Bolivian Chaco, the prevalence of lifetime epilepsy was 12.3/1000 [34]. Recently, the lifetime prevalence of epilepsy associated with generalized tonic-clonic seizures was found to be 7.2/1000, with a crude incidence risk of 55.4/100,000 in the same area [35]. During the prevalence survey carried out in 1994, we also evaluated the prevalence of NCC among people with epilepsy (PWE) identified in the Bolivian Chaco. Considering the laboratory findings and radiological and clinical features, 27.4% of PWE fulfilled the Del Brutto diagnostic criteria [36] for definitive or probable NCC, and 70.6% of these patients presented with focal seizures with or without a secondary generalization [32]. This was one of the first pieces of evidence that around 30% of PWE in *T. solium* endemic areas were affected by NCC and that it was mainly associated with late-onset focal epilepsy. These findings were largely confirmed by several studies successively carried out in Latin American countries during the last decades [35].

Other studies in the Bolivian Chaco have also highlighted the possible association between epilepsy and toxocariasis [37]. Human toxocariasis is a zoonosis caused by the larval stage of the parasite *Toxocara canis* and, less frequently, *Toxocara cati*, which generally affects dogs and cats. Human toxocariasis is one of the most widespread helminthiases in the world whenever the human–soil–dog relation is particularly close, especially in LMIC. Visceral larva migrans and ocular larva migrans are the most frequent clinical manifestations, although most infections remain undiagnosed due to being asymptomatic [38,39]. *Toxocara* larvae can pass through the blood–brain barrier and invade the central nervous system, resulting in disease. A population-based case–control study conducted in the Bolivian Chaco showed a positive association between *T. canis* seropositivity and epilepsy with an OR of 2.70 (95% CI 1.41–5.19), which was stronger when the analysis was restricted to late-onset focal epilepsy [37]. Other case–control studies have been carried out over time in different countries and different settings (high-income countries and LMIC), but this association is still being debated although confirmed by two different meta-analyses [40].

In LMIC, there are significant disparities in the care provided to epileptic patients. According to a recent meta-analysis in Latin American countries, the treatment gap (TG) is 60.6%, with high differences between rural and urban areas (77.8% versus 26.2%) [41]. In the Bolivian Chaco, a TG of 90% was recorded in 1994 [34]. Epidemiology and the management of epilepsy in the Bolivian Chaco have been addressed over the last 25 years. In particular, to improve knowledge about epilepsy and to reduce the epilepsy TG, many training courses directed at health workers (both GPs and community health workers), as well as awareness campaigns towards rural populations, have been organized in the communities of the Bolivian Chaco [42,43]. According to a recent study, an improvement in epilepsy knowledge, awareness, and practices in these areas was recorded, leading to a reduction of the TG over time from 90% to 45% [43]. On the basis of these findings, educational campaigns were shown to be an important tool to reduce the epilepsy TG in LMIC.

## 8. Trichinellosis

*Trichinella* spp. are one of the most widespread parasites infecting people and other mammals all over the world in several climates, except for deserts [44]. Humans can acquire the parasite by eating uncooked or inadequately treated meat from domestic or wild animals [45].

Trichinellosis was never reported in Bolivia until 1991, when the first report was confirmed in swine from the Bolivian Altiplano [46]. Since then, in the Bolivian Chaco, a large seroprevalence study was conducted on 1327 swine sera, of which 13.4% were found to be positive [47]. In humans, a serosurvey performed on 234 sera in Santa Cruz Province showed the presence of antibodies against *T. spiralis* in 3% of the patients [48]. Furthermore, in 2011, swine muscular samples (*n* = 65) from a slaughterhouse located in the department of Santa Cruz were examined using the artificial digestion method and serology. No larvae were detected, but 6 out of the 255 serum samples were positive (2.3%) [49].

## 9. Discussion and Conclusions

The long-standing research and cooperation activities carried out by Bolivian and Italian teams for over 35 years due to an official agreement signed in 1987 between the Bolivian MoH and the University of Florence (Italy) allow an increase in knowledge and awareness about several neglected parasitic diseases that represent a public health concern in the Bolivian Chaco. The results achieved by the 35-year collaboration informed public health authorities about the burden and dynamics of these diseases and allowed targeted public health interventions (epilepsy treatment; Water, Sanitation, and Hygiene—WASH program; and other educational campaigns) in rural areas. Capacity-building activities at the local level allow for the strengthening of the skills required to diagnose, manage, and evaluate local sanitary problems, as well as the foundation of a collaborative network between Italian and Bolivian researchers.

The monitoring of STHs after a mass deworming campaign allow the interruption of PC delivery in the Bolivian Chaco. Surveillance studies were performed yearly at the sentinel sites until 2019 and confirmed a persistent low prevalence of STHs despite PC interruption (unpublished data).

After being forced to stop for two years owing to the COVID-19 epidemic, STH monitoring is ready to start up again. Our findings suggest that helminths and fecal–oral protozoa should be given more attention as emerging pathogens, particularly in regions where STHs are successfully controlled and other parasite illnesses that were formerly associated with anemia have decreased. WASH programs are essential for improving these populations’ health issues, particularly anemia, in a sustained way, as well as the promotion of training courses and health education as a key tool to prevent the transmission of several pathogens. The effects of a school-based health education intervention on handwashing in children involving SAC were recently demonstrated [50], showing a significant increase in hand-washing behaviors, but further actions should be promoted in the area in order to improve the state of health of the populations involved.

Studies on Chagas diseases confirmed that vectorial *T. cruzi* transmission persists in the Bolivian Chaco. Recent progress in the field of Chagas disease diagnostics and treatment paves the way toward improved access to care for people living in endemic remote areas through the implementation of screen-and-treat programs and a telemedicine-based approach. The use of two combined rapid diagnostic tests has proven to be a reliable and accurate alternative to conventional serological assays to achieve a conclusive diagnosis of Chagas disease, thus facilitating access to early diagnosis in deprived settings, where equipped labs and trained personnel are not available [51]. Point-of-care molecular-based methodologies have been tested, showing promising results for the early diagnosis of congenital Chagas disease [52]. Moreover, the mobile health-based strategy proved to be effective in identifying patients with signs of early cardiomyopathy who may benefit from a further cardiologic assessment to limit disease progression and morbidity [53]. Finally, the BENDITA phase II trial suggested that shorter courses of benznidazole may provide an equivalent parasitological efficacy in the current 60-day standard course, opening the door to large-scale anti-trypanosomal treatment [54]. However, all these advances may be vain if they are not accompanied by a successful campaign for the control of domestic *T. infestans* and the elimination of *T. cruzi* vectorial transmission.

The few studies conducted on animals as reservoirs of zoonotic parasites, as well as of Chagas disease and filariasis, showed the important role of animals in the life cycles of these parasites and underlined the importance of the One Health concept in defining the epidemiological scenario of such infections. Therefore, to evaluate the potential animal reservoirs, future studies have been planned to collect samples from different species of animals living in the communities in close contact with the population.

The unexpected finding of *B. microti* in healthy people stimulates the interest in the study of tick-borne diseases in the area, as only another study was performed on babesiosis in cattle in the Bolivian Chaco. Extensive collections of ticks or other biological vectors of parasites are warranted to better characterize the epidemiology of the vector-borne diseases in the area and to plan more efficient control programs. The presence of tick-borne borreliosis was firstly reported in the Bolivian Chaco in both humans and animals in the 1990s [55,56].

Studies on trichinellosis in humans and pigs [48,49] highlight the need to strengthen the surveillance of trichinellosis in municipal slaughterhouses and to prevent the clandestine slaughter of animals. The prevention of human trichinellosis through adequate meat inspection is a classic achievement of veterinary public health measures. Of fundamental importance for health improvement is also to inform the local authorities about the health risk and the prevention measures. In this regard, local authorities in the Department of Santa Cruz were trained on techniques for detecting *Trichinella* larvae with artificial digestion, as there were no regular slaughter controls for this parasitosis. (personal communication Macchioni).

The participation of the communities was a key factor in the success of the research activities. The support of the Asamblea del Pueblo Guaraní, the Guaraní political organization, from the start of the research programs was of paramount importance to approach the communities and inform the community leaders and other members, to know the community perception, to obtain additional information, and to ensure logistic support. The full support of the communities, as well as of the families, students, teachers, and health personnel, allowed for the long-term sustainability of the research programs. The students of the Escuela de Salud del Chaco Tekove Katu of Gutierrez (Cordillera Province), supervised by expert health personnel, provided important support in the fieldwork, including the administration of questionnaires, collection of samples, etc. The parasitological procedures have been performed by Bolivian and Italian research team members, partly in the laboratories of the local hospitals and partly in Italy.

Since building an effective capacity at the local level is crucial to strive with new local health challenges and emerging diseases, further activities to improve the knowledge on infectious diseases, including parasitosis, will be promoted involving sanitary personnel from other regions.

In conclusion, the results obtained through a consolidated international research activity between Bolivian and Italian teams in the Bolivian Chaco represent an example of south–north and north–south fruitful collaborations. Moreover, the continuous research activities in the field of parasitology allowed a privileged point of view about the epidemiologic trend of several otherwise neglected conditions that still represent major health concerns in this remote area. The findings obtained within this collaboration, which involved a huge number of local and international partners over the years, laid the basis for future interventions and further research and provided useful information to address not only local but also global health challenges, shared by several deprived regions from LMIC.

## Figures and Tables

**Table 1:** Seroprevalence for IgG anti-*Trypanosoma cruzi* in the human population of the surveyed localities in 1987, 1997, 2006, and 2013.

Year/Study Area	Overall Prevalence (%)
1987	
Camiri	72.5
Boyuibe	63.6
Javillo	97.6
1997	
Camiri	81.1
Villa Montes	73.6
2006	
Bartolo	69.6
2013	
Ivamirapinta	60.3
Bartolo	63.3

## Data Availability

Not applicable.
